# Post-migration acquisition of HIV: Estimates from four European countries, 2007 to 2016

**DOI:** 10.2807/1560-7917.ES.2021.26.33.2000161

**Published:** 2021-08-19

**Authors:** Zheng Yin, Alison E Brown, Brian D Rice, Gaetano Marrone, Anders Sönnerborg, Barbara Suligoi, Andre Sasse, Dominique Van Beckhoven, Teymur Noori, Vincenza Regine, Valerie C Delpech

**Affiliations:** 1 HIV and STI Department, CIDSC, Public Health England, Colindale, London, United Kingdom; 2 Faculty of Public Health and Policy, London School of Hygiene and Tropical Medicine, London, United Kingdom; 3 Department of Infectious Diseases, Karolinska Institutet, Karolinska University Hospital, Stockholm, Sweden; 4 National AIDS Unit, Department of Infectious Diseases, National Institute of Health, Rome, Italy; 5 Scientific Institute of Public Health, Brussels, Belgium; 6 European Centre for Disease Prevention and Control, Stockholm, Sweden

**Keywords:** HIV migrants Europe, Europe, HIV infection

## Abstract

**Background:**

The assumption that migrants acquire human immunodeficiency virus (HIV) before migration, particularly those from high prevalence areas, is common.

**Aim:**

We assessed the place of HIV acquisition of migrants diagnosed in four European countries using surveillance data.

**Methods:**

Using CD4^+^ T-cell count trajectories modelled to account for seroconversion bias, we estimated infection year of newly HIV-diagnosed migrants residing in the United Kingdom (UK), Belgium, Sweden and Italy with a known arrival year and CD4^+^ T-cell count at diagnosis. Multivariate analyses identified predictors for post-migration acquisition.

**Results:**

Between 2007 and 2016, migrants constituted 56% of people newly diagnosed with HIV in the UK, 62% in Belgium, 72% in Sweden and 29% in Italy. Of 23,595 migrants included, 60% were born in Africa and 70% acquired HIV heterosexually. An estimated 9,400 migrants (40%; interquartile range (IQR): 34–59) probably acquired HIV post-migration. This proportion was similar by risk group, sex and region of birth. Time since migration was a strong predictor of post-migration HIV acquisition: 91% (IQR: 87–95) among those arriving 10 or more years prior to diagnosis; 30% (IQR: 21–37) among those 1–5 years prior. Younger age at arrival was a predictor: 15–18 years (81%; IQR: 74–86), 19–25 years (53%; IQR: 45–63), 26–35 years (37%; IQR: 30–46) and 36 years and older (25%; IQR: 21–33).

**Conclusions:**

Migrants, regardless of origin, sex and exposure to HIV are at risk of acquiring HIV post-migration to Europe. Alongside accessible HIV testing, prevention activities must target migrant communities.

## Introduction

Ca 22.3 million (4.4%) people living in the European Union (EU) today are non-EU citizens, of whom an estimated 2.4 million migrated to the EU in 2017 alone [1]. While most migrants are human immunodeficiency virus (HIV)-negative [2], a minority of migrants may be more vulnerable to HIV [3] because of an elevated prevalence in their country of origin. The European Centre for Disease Prevention and Control (ECDC) reported that more than two in five people diagnosed with HIV in 2018 in the EU/European Economic Area (EEA) were born outside the country in which they were diagnosed, with considerable variation across Europe [4]. It is often assumed migrants acquired HIV in their country of origin [5], particularly if prevalence is elevated in these settings. There is, however, growing evidence that people are at continued risk of acquiring HIV post-migration [6]. This is mainly because sexual networks of migrants within destination countries may be largely confined to others from within the same community [6].

Understanding the probable timing of HIV acquisition in relation to migration is important to inform national prevention initiatives [7], but poses challenges. Previous studies attempted to assign a probable place and time of infection based upon assessment of self-reported sexual history at diagnosis [5,8]. In isolation, these assessments are ambiguous because a person can have an undiagnosed HIV infection and/or may have multiple potential HIV exposures over many years. Furthermore, an individual may be unwilling to share or unable to remember their sexual behaviours, leading to uncertainties and recall biases [5,6].

To provide a population level estimate of post-migration acquisition and to address the ambiguities using clinical history, Public Health England (PHE) developed a novel method that applied routinely collected surveillance parameters (country of birth, year of arrival and CD4^+^ T-cell count at diagnosis) to averaged time-modelled slopes of CD4^+^ T-cell decline. Using this method, Rice et al. estimated that 33% of heterosexuals born outside the United Kingdom (UK) and diagnosed with HIV between 2004 and 2010 acquired HIV after arrival into the UK [9], a figure three times greater than that based on clinical assessment. Similarly, Desgrees-du-Loû et al. applied a mixed method, including both live-event questionnaires and modelling the CD4^+^ T-cell decline, to assign post-migration acquisition among Sub-Saharan Africans living in France [10]. The advancing migrant access to health services in Europe (aMASE) study used a similar approach combined with qualitative methods and estimated that 63% of migrants acquired infection post-migration across nine countries in Europe [11].

In the aforementioned studies, CD4^+^ T-cell trajectories were derived from the averages of multiple CD4^+^ T-cell counts taken from individuals for whom the seroconversion date was approximately known. Then, the CD4^+^ T-cell count at diagnosis was applied against the modelled slopes to estimate infection length. However, CD4^+^ T-cell counts may temporarily decline during seroconversion [12], typically around 6 to 8 weeks following exposure. Thus, CD4^+^ T-cell trajectories that include counts taken during seroconversion would flatten the slopes, underestimating the extent that migrants acquired HIV post-migration.

Here we estimate and compare the proportion of post-migration HIV acquisitions within migrant communities diagnosed in four European countries (UK, Belgium, Sweden and Italy) using routinely collected surveillance data. We further refine PHE’s method to account for the bias from inclusion of samples taken from seroconverting individuals in the modelled CD4^+^ T-cell trajectories. A multivariate regression analysis is undertaken to identify predictors for post-migration HIV acquisition overall and among those diagnosed 1 to 5 years after migration.

## Methods

### Study population

The ECDC HIV surveillance network invited all EU/EEA countries with HIV surveillance systems containing the required data fields to participate; the UK, Belgium, Sweden and Italy agreed. A 2-day workshop was held at PHE to discuss the project objectives. and agree on the final methodology before data collection and analyses.

Relevant surveillance information on all people diagnosed with HIV between 2007 and 2016 and reported to national HIV surveillance systems in the UK, Belgium, Sweden and Italy were gathered and analysed at PHE. All four countries provided the following: date of HIV diagnosis, CD4^+^ T-cell count at diagnosis (or within 91 days of diagnosis), treatment status (treatment naive or treatment start date, to ensure CD4^+^ T-cell counts used in the analysis were extracted when people were treatment naive), age at diagnosis, sex and year of arrival into the destination country, country of birth and exposure category. Details of the national HIV surveillance systems in these countries have previously been described [13-18]. In our analyses, migrants were defined as individuals aged 15 years and older, born outside the country of diagnosis. Migrants were included if they had complete information on year of arrival in their destination country and a CD4^+^ T-cell count within 91 days of diagnosis.

To model CD4^+^ T-cell slopes following seroconversion, a separate dataset was created from the Swedish and UK surveillance data collections (this information was not available in Italy or Belgium). This dataset included 1,653 people aged 15 years and older, irrespective of migrant status, who were diagnosed between 2000 and 2014 with evidence of seroconversion (defined as a documented negative HIV test within 1 year of HIV diagnosis and at least two CD4^+^ T-cell measurements before starting treatment and/or death), as outlined in the Supplement.

### Estimation of seroconversion time with CD4^+^ T-cell trajectories

CD4^+^ T-cell slope trajectories (referred to as ‘the slope’) for people with known last negative and first positive HIV tests were built on the square root of CD4^+^ T-cell counts modelled through a maximum likelihood multilevel linear approach [19-23]. Since people in this cohort might present at the time of seroconversion, an ‘anchor’ date 3 months after HIV diagnosis was set as the CD4^+^ T-cell count (where the person remained treatment-naive) to remove the bias of capturing a seroconversion-induced low CD4^+^ T-cell count (Supplement). Unlike previous studies that use the mid-point of HIV-negative and -positive test dates, we modelled the slopes using counts reported from the anchor date onwards. The estimated CD4^+^ T-cell count at the anchor date was taken as the ‘intercept’. The follow-up period for each seroconverted individual was restricted to the point of treatment initiation, date of death or a 10-year period following diagnosis (for treatment naive people).

Potential predictors that could affect CD4^+^ T-cell trajectories were identified as: sex, age, region of birth (Europe, Africa or other) and HIV exposure (men who have sex with men (MSM) contact, heterosexual contact and other). For each predictor identified in a multivariable linear model, a specific CD4^+^ T-cell trajectory (slope, intercept and interquartile range (IQR)) was calculated. Full methods and results from this model are detailed in the Supplement. The median time between the anchor date and the first CD4^+^ T-cell count was calculated as follows:

*t-median* (probable median interval between the anchor date and first CD4^+^ T-cell count)

$$=\frac{\sqrt{M}-\sqrt{first\;CD4\;cell\;count}}{S}$$

where *M* is the median of the intercept, and *S* is the slope.

Final formulae were adjusted for age group and region of birth subgroup (three groups were defined: Europe, Africa and other) to consider of the predictors associated with the slope and intercept (Supplement). The probable time of HIV infection for each person was then estimated to be t-median years before the first CD4^+^ T-cell count (IQR: +0.25 to +1.25 years around the estimated median time of HIV seroconversion).

### Assigning post-migration acquisition

For each newly diagnosed individual, a period of HIV infection (defined as the IQR around the median) was estimated through the CD4^+^ T-cell trajectory formula using CD4^+^ T-cell count, age at diagnosis, and region of birth (formulae 1–3 in the Supplement). Each person was then classified into one of three categories: ‘pre-migration’ HIV acquisition, ‘post-migration’ HIV acquisition or ‘undetermined’. Classification was done according to whether the period of HIV infection occurred before, after or within their reported year of arrival, respectively. Where there was ambiguity resulting from IQR, we assumed that people with a CD4^+^ T-cell count higher than the upper quartile limit belonged to the corresponding category ‘post-migration’ HIV acquisition; people with a CD4^+^ T-cell count lower than the lower quartile limit were assumed to have acquired infection pre-migration. At the population level, upper and lower estimates of pre- and post-migration acquisition were generated; central estimates were produced through reassigning all ‘undetermined’ to either ‘post-‘ or ‘pre-migration’ based upon relative positioning of the median intercept. Migrants whose year of infection was the same as year of arrival were allocated as having acquired infection pre-migration. Where presented, years relate to year of diagnosis, and not year of infection.

### Multivariate and statistical analyses

Predictors for acquiring HIV after migration were identified through logistic regression analyses. Factors that were found significant in univariate models were included in a multivariate model. An additional analysis was undertaken to identify predictors for those acquiring HIV infection post-migration and had arrived 1 to 5 years before HIV diagnosis.

Continuous variables were compared using the Wilcoxon–Mann–Whitney test. Rates were compared using the Pearson’s chi-squared test. The Cochran–Armitage chi-squared trend test was used for time trend analyses. Bivariate and multivariable multilevel regression models were used to identify significant risk factors. Testing values and confidence intervals (CI) are at the 95% significance level. STATA (v13.0, StataCorp, College Station, Texas, United States) was used for analyses.

### Ethical statement

Ethical approval was not needed since all information in the study was collected through routine national HIV surveillance.

## Results

### Modelling of CD4^+^ T-cell trajectories to estimate seroconversion

Overall, 1,653 people (≥ 15 years) with the last negative test and HIV diagnosis dates within one year (1,233 from the UK and 420 from Sweden) were included in the CD4^+^ T-cell modelling, comprising a collective 15,881 CD4^+^ T-cell counts (Supplement). Most of the 1,653 people were MSM (81%) and born in Europe (84%), and two-thirds of people were diagnosed after 2005. The median interval between the last negative test and HIV diagnosis was 191 days (IQR: 105–271), and 11% of seroconverted individuals had a test interval of under 2 months. The median time between HIV diagnosis and the first CD4^+^ T-cell count after the anchor date was 141 days (IQR: 115–187); the median interval from the anchor date to end of follow-up was 3.7 years (IQR: 1.5–7.0).

The median CD4^+^ T-cell count at the anchor date differed significantly by region of birth (p < 0.001); counts were 561, 438 and 543 cells/µL among those born in Europe, Africa and elsewhere, respectively. There was no relationship with age at diagnosis. Full results are provided in the Supplement.

The average decline in CD4^+^ T-cell count in the first year following seroconversion was 46, 65 and 67 cells/µL among those born in Europe, Africa and elsewhere, respectively. Each increase in year of age was associated with a 1.3% increase in the rate of CD4^+^ T-cell decline (p = 0.002). Predictors were adjusted for in the final formulae (Supplement). No significant differences between MSM and heterosexual men and women were observed.

### Population characteristics of HIV-diagnosed migrants

Migrants constituted 56% of people newly diagnosed with HIV in the UK, 62% in Belgium, 72% in Sweden and 29% in Italy between 2007 and 2016 (Table 1). Overall, 49% (23,595/48,314) of migrants diagnosed with HIV had available data and were included in our analyses. Completeness of information varied by country; the percentages of compete data from the UK, Belgium, Sweden and Italy were 54% (17,856/32,892), 53% (2,635/4,979), 59% (1,634/2,785) and 19% (1,470/7,658), respectively.

**Table 1:** Migrants diagnosed with HIV in the United Kingdom, Belgium, Sweden and Italy, 2007–2016 (n = 23,595)

Population characteristics	UK		Belgium		Sweden		Italy	
Year of HIV diagnosis	2007–16		2007–16		2007–16		2010–16	
All HIV diagnoses (n, ≥ 15 years)	63,309		10,511		3,957		26,643	
Migrant population	n	%	n	%	n	%	n	%
Total observed	32,892		4,979		2,785		7,658	
Country of birth available	30,261	92	3,832	77	2,729	98	7,658	100
Reported CD4^+^ T-cell count at diagnosis	30,964	94	3,368	68	2,762	99	5,413	32
Reported year of arrival	21,888	66	3,734	75	1,653	59	1,543	20
Total eligible^a^	17,856	54	2,635	53	1,634	59	1,470	19

HIV: human immunodeficiency virus; UK: United Kingdom

^a^ Migrants eligible for this study had complete information on CD4^+^ T-cell count at HIV diagnosis and year of arrival.

In all four countries, ca 70% of the study population (16,517/23,595) was aged 30 years and above at diagnosis, and 68% (16,045) acquired HIV through heterosexual contact. Over 60% were African-born and more than half were diagnosed late (CD4^+^ T-cell count < 350 cells/µl) in the UK, Sweden, and Italy with 47% in Belgium (Table 2). The age distribution and CD4^+^ T-cell count at diagnosis among migrants included and excluded was broadly similar, however, migrant MSM, migrants from within the EU, and those who migrated more recently were slightly less likely to be included (Supplement).

**Table 2:** Study population characteristics of migrants diagnosed with HIV infection in the United Kingdom, Belgium, Sweden and Italy, 2007–2016 (n = 23,595)

Characteristics		UK		Belgium		Sweden		Italy	
		n	%	n	%	n	%	n	%
Total eligible migrants		17,856		2,635		1,634		1,470	
Age at diagnosis (years)	15–30	5,306	30	764	29	506	31	550	37
	31–50	10,839	61	1,610	61	1,009	62	810	55
	≥ 51	1,711	10	261	10	119	7	110	7
Age at arrival (years)	15–18	1,197	7	126	5	104	6	94	6
	19–25	4,681	26	540	20	307	19	410	28
	26–35	7,327	41	1,061	40	702	43	575	39
	≥ 36	4,651	26	908	34	521	32	391	27
Exposure group	MSM	4,767	27	699	27	283	17	197	13
	Het men	4,448	25	580	22	356	22	429	29
	Het women	7,629	43	1,123	43	709	43	684	47
	PWID men	277	2	34	1	28	2	48	3
	PWID women	82	0	10	0	4	0	14	1
	Blood-related products	274	2	102	4	20	1	18	1
First CD4+ T-cell count (cells/µl)	< 200	5,746	32	649	25	372	23	584	40
	200–349	4,024	23	591	22	442	27	310	21
	350–499	3,470	19	574	22	335	21	261	18
	≥ 500	4,616	26	821	31	485	30	315	21
Region of birth	Eastern Europe	1,225	7	136	5	57	3	244	17
	Northern Europe	635	4	15	1	44	3	5	0
	Southern Europe	1,342	8	159	6	46	3	70	5
	Western Europe	462	3	221	8	16	1	6	0
	Eastern Africa	5,733	32	368	14	701	43	57	4
	Middle Africa	686	4	644	25	109	7	87	6
	Northern Africa	105	1	77	3	23	1	83	6
	Southern Africa	1,043	6	19	1	20	1	5	0
	Western Africa	2,978	17	539	21	173	11	665	45
	Latin America and Caribbean	1,517	8	232	9	82	5	188	13
	Asia-Pacific	1,894	11	194	7	355	22	59	4
	North America	236	1	20	1	8	0	0	0
Year of arrival	< 1983	419	2	51	2	48	3	16	1
	1983–95	1,164	7	124	5	100	6	65	4
	1996–99	1,159	6	76	3	25	2	73	5
	2000–04	4,981	28	297	11	111	7	189	13
	2005–09	5,113	29	777	29	483	30	292	20
	2010–16	5,020	28	1,310	50	867	53	835	57
Year of diagnosis	2007	1,983	11	184	7	64	4	0	0
	2008	2,020	11	214	8	90	6	0	0
	2009	2,010	11	229	9	193	12	0	0
	2010	1,895	11	298	11	219	13	123	8
	2011	1,775	10	325	12	197	12	236	16
	2012	1,588	9	314	12	174	11	272	19
	2013	1,510	8	300	11	175	11	168	11
	2014	1,703	10	257	10	216	13	204	14
	2015	1,854	10	259	10	157	10	181	12
	2016	1,518	9	255	10	149	9	286	19
Time between arrival and HIV diagnosis	Same year	3,667	21	1,023	39	740	45	554	38
	1–5 years	6,353	36	998	38	556	34	456	31
	6–10 years	4,265	24	298	11	145	9	205	14
	> 10 years	3,571	20	316	12	193	12	255	17
Median age at diagnosis, years (IQR)		35 (23–43)		35 (29–43)		35 (29–41)		33 (27–41)	
Median age at arrival, years (IQR)		29 (24–36)		31 (25–38)		31 (25–38)		29 (23–36)	
Median time between arrival and diagnosis, years (IQR)		4 (1–9)		1 (0–5)		1 (0–5)		2 (0–8)	
Median CD4+ T-cell count at diagnosis, cells/µl (IQR)		318 (148–507)		367 (201–557)		355 (205–534)		271 (100–458)	

HIV: human immunodeficiency virus; Het: heterosexual; IQR: interquartile range; MSM: men who have sex with men; PWID: people who inject drugs; UK: United Kingdom.

Median CD4^+^ T-cell count taken within 91 days of HIV diagnosis.

### Estimation of post-migration HIV acquisition

An estimated 40% (IQR: 34–59) of migrants acquired HIV post-migration. Of these, a similar proportion were European-born (40%; IQR: 36–49) or African-born (39%; IQR: 32–48). This proportion was slightly higher among migrants born in Latin America and the Caribbean (48%; IQR: 43–56; Table 3). A similar proportion of MSM (45%; IQR: 40–53) and heterosexual men (42%; IQR: 36–51) acquired infection post-migration. This proportion was slightly lower among heterosexual women (38%; IQR: 31–47). The highest percentage of migrants who acquired HIV post-migration was among those who had lived in the country of destination for ≥ 10 years (91%; IQR: 87–95), compared with 63% (IQR: 51–73) and 30% (IQR: 21–37) among those who arrived between 6–10 years and 1–5 years ago, respectively. A higher proportion of younger people (age at arrival) were estimated to have acquired the HIV infection in their country of destination: 15–18 years (81%; IQR: 74–86); 19–25 years (53%; IQR: 45–63); 26–35 years (37%; IQR: 30–46); 36 years and older (25%; IQR: 21–33) (Table 3). If the 5,985 individuals whose estimated year of HIV acquisition was the same as their year of arrival were instead categorised as ‘post-migration HIV acquisition’, the proportion would increase from 40% to 65%.

**Table 3:** Predictors of post-migration HIV acquisition among migrants diagnosed in the United Kingdom, Belgium, Sweden and Italy, 2007–2016 (n = 23,595)

		Migrants (n)	Post-migration HIV acquisition % (IQR)	aOR (95% CI)
Total eligible		23,595	40 (34–59)	–
Age at diagnosis^a ^(years)	15–30	7,126	33 (26–43)	NI
	31–50	14,268	42 (36–51)	NI
	≥ 51	2,201	56 (52–61)	NI
Age at arrival (years)	15–18	1,521	81 (74–86)	2.63 (2.18–3.15)
	19–25	5,938	53 (45–64)	1.59 (1.44–1.76)
	26–35	9,665	37 (30–45)	1.10 (1.01–1.20)
	≥ 36	6,471	25 (21–49)	Ref.
Exposure group	MSM	5,946	45 (41–53)	1.71 (1.53–1.92)
	Het men	5,813	42 (36–51)	0.85 (0.78–0.93)
	Het women	10,145	38 (31–47)	Ref.
	PWID men	387	30 (27–38)	0.94 (0.71–1.24)
	PWID women	110	31 (25–38)	0.80 (0.60–1.06)
	Blood related products	414	30 (27–37)	0.81 (0.64–1.02)
First CD4^+^ T-cell count^b^ (cells/µl)	< 200	7,351	20 (15–27)	NI
	200–349	5,367	37 (28–49)	NI
	350–499	4,640	51 (41–65)	NI
	≥ 500	6,237	60 (57–63)	NI
Region of birth	Eastern Europe	1,662	36 (31–46)	1.4 (1.14–1.72)
	Northern Europe	699	46 (41–55)	1.65 (1.27–2.15)
	Southern Europe	1,617	38 (34–46)	1.34 (1.07–1.66)
	Western Europe	705	47 (43–55)	1.72 (1.31–2.26)
	Eastern Africa	6,859	41 (33–50)	1.12 (0.95–1.31)
	Middle Africa	1,526	29 (24–37)	Ref.
	Northern Africa	288	50 (42–56)	1.71 (1.17–2.48)
	Southern Africa	1,087	43 (36–52)	1.52 (1.23–1.89)
	Western Africa	4,355	38 (31–46)	1.31 (1.1–1.55)
	Latin America and Caribbean	2,019	48 (43–56)	1.43 (1.17–1.76)
	Asia-Pacific	2,502	43 (38–52)	1.14 (0.94–1.38)
	Northern America	264	35 (33–41)	1.25 (0.82–1.90)
Year of arrival	< 1983	534	100 (no range)	NI^c^
	1983–1995	1,453	97 (95–99)	NI
	1996–1999	1,333	80 (73–87)	NI
	2000–2004	5,578	64 (52–74)	NI
	2005–2009	6,665	31 (24 –42)	NI
	2010–2016	8,032	11 (8–18)	NI
Year of diagnosis	2007	2,231	37 (29–48)	NI^c^
	2008	2,324	38 (30–47)	NI
	2009	2,432	40 (32–50)	NI
	2010	2,535	43 (37–52)	NI
	2011	2,533	41 (35–50)	NI
	2012	2,348	41 (40–54)	NI
	2013	2,153	45 (38–51)	NI
	2014	2,380	44 (38–51)	NI
	2015	2,451	38 (33–43)	NI
	2016	2,208	36 (32–43)	NI
Time between arrival and HIV diagnosis^d^	Same year	5,984	0 (no range)	NA
	1–5 years	8,363	30 (21–46)	Ref.
	6–10 years	4,913	63 (51–73)	4.23 (3.90–4.58)
	˃ 10 years	4,335	91 (87–95)	23.11 (20.49–26.07)

aOR: adjusted odds ratio; HIV: human immunodeficiency virus; Het: heterosexual; IQR: interquartile range; MSM: men who have sex with men; NA: not available; NI: not included in regression analyses; PWID: people who inject drugs; Ref: reference.

^a^ We used age at arrival in the multivariate regression model.

^b^ First CD4^+^ T-cell count at diagnoses was not included in the regression model as it was not considered as a potential predictor for post-migration acquisition.

^c^ Year of arrival and year of diagnosis were not included. Instead, we included the time between arrival and diagnosis.

^d^ We assumed that people who were diagnosed in the same year of arrival acquired infection pre-migration.

#### United Kingdom

Overall, 44% (7,857/17,856; IQR: 37–53) of the migrants diagnosed between 2007 and 2016 had acquired HIV post-migration, increasing from 38% (754/1,983; IQR: 30–49) in 2007 to 49% (826/1,703; IQR: 42–56) in 2014. The proportion dropped slightly to 42% (782/1,854; IQR: 37–50) in 2015 and 41% (635/1,518; IQR: 38–49) in 2016 (10-year trend analysis: p < 0.05) (Figure).

**Figure:**
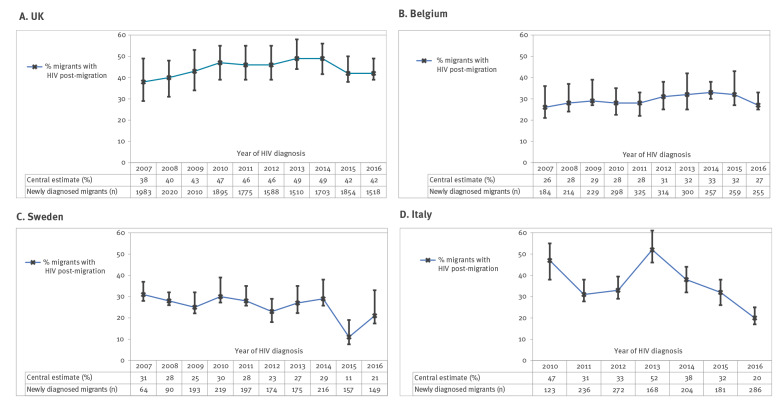
Proportion of migrants who acquired HIV post-migration by destination country and year of diagnosis, United Kingdom, Belgium, Sweden, Italy, 2007–2016 (n = 23,595)

HIV: human immunodeficiency virus; UK: United Kingdom

From 2007–16, 45% (2,150/4,767; IQR: 41–53) of migrants who were MSM probably acquired HIV post-migration. The absolute number (not adjusted for excluded migrants) more than doubled from 135 (IQR: 123–174) in 2007 to 288 (IQR: 260–341) in 2015, while the percentage increased from 42% (IQR: 39–55) in 2007 to 51% (IQR: 45–58) in 2011 and dropped to 38% (IQR: 35–45) in 2015. An estimated 228 MSM migrants to the UK probably acquired HIV post-migration in 2016. Overall, the proportion of heterosexual migrants who acquired HIV post-migration was 44% (IQR: 36–53) rising from 37% (IQR: 27–48) in 2007 to 47% in 2015 and 2016. The number of heterosexual migrants who acquired HIV pre-migration decreased (1,586 in 2007 to 449 and 358 in 2015 and 2016, respectively).

#### Belgium

An estimated 29% (764/2,635; IQR: 25–37) migrants acquired HIV post-migration, ranging between 26–31% over the period (Figure). The percentage and number of heterosexual migrants who acquired HIV post-migration increased from 26% (47/181) in 2007 to 32% (95/297) in 2013 and dropped slightly to 27% (70/259) in 2016 (p value = 0.78). Among MSM migrants, the number (not adjusted for migrants excluded from the analysis) rose from 11 to 33 with a peak of 45 in 2013 and the corresponding percentage changed from 38% in 2007 to 45% in 2013 and back to 38% in 2016 (p = 0.627).

#### Sweden

Overall, an estimated 25% (409/1,634; IQR: 22–33) migrants acquired HIV post-migration. Half of MSM migrants (138/283; IQR: 44–57) and 22% (239/1,065; IQR: 19–31) of heterosexual migrants acquired infection post-migration with no clear time trend.

#### Italy

An estimated 34% (IQR: 29–40) of 1,470 migrants acquired HIV post-migration. More than half of MSM migrants (111/197; 56%, IQR: 49–65) and 31% (342/1,113; IQR: 26–37) of heterosexual migrants acquired infection post-migration with no clear time trend.

### Predictors for post-migration HIV acquisition

We included the following predictors in a bivariate analysis for post-migration HIV acquisition: age at arrival, risk-group, country of birth, year of arrival, year of diagnosis and time between migration and diagnosis.

Table 3 summarises the adjusted odds ratios (aOR) associated with post-migration HIV acquisition. People aged 15–18 years at arrival were more likely to have acquired HIV post-migration compared with those aged 36 years and older (aOR: 2.63; 95% CI: 2.18–3.15). MSM were more likely to acquire HIV post-migration (aOR: 1.71; 95% CI: 1.53–1.92) compared with (aOR: 0.85; 95% CI: 0.78–0.93) heterosexual men. People who arrived 10 years or more before diagnosis were 23 times more likely to acquire HIV post-migration (aOR: 23.11; 95% CI: 20.49–26.07) compared with people who arrived within 5 years before diagnosis.

In a specific multivariate analysis of migrants who had recently arrived (1–5 years before diagnosis; 30% of study population), predictors of post-migration acquisition included age at arrival (15–18 years (aOR: 2.43; 95% CI: 1.80–3.28); 19–25 years (aOR: 1.63; 95% CI: 1.43–1.86); 26–35 years (aOR: 1.18; 95% CI: 1.05–1.33); reference category: ≥ 36 years and sex between men (aOR: 1.63; 95% CI: 1.41–1.89) reference category: heterosexual women).

## Discussion

We present multi-country estimates of post-migration HIV acquisition among 23,595 migrants diagnosed between 2007 and 2016 in Europe using a model parametrised by routinely collected surveillance data and adjusted for the potential effect of seroconversion. An estimated two in five migrants diagnosed with HIV in four European countries (UK, Belgium, Sweden and Italy) acquired HIV post-migration. While this proportion increased with a longer time spent in the country of destination before HIV diagnosis, one in three migrants acquired HIV between 1 and 5 years before diagnosis after migration occurred. Our findings indicate that the public health response to HIV among migrant communities within Europe should be expanded from HIV testing strategies to also include primary prevention, targeting populations who have arrived relatively recently.

The ongoing risk of HIV acquisition after arrival among migrants from high HIV prevalence settings is perpetuated by different factors. Regardless of country of origin, sexually active migrants are most likely to mix within their own communities [6]. For others, migration may change sexual behaviours. Some MSM may choose to migrate due to restrictive societal attitudes towards gay communities in their country of origin [24]. Nevertheless, migration to western Europe where HIV prevalence among the MSM community is high could indicate that migrant MSM may be at risk of HIV acquisition post-migration. It is important to note that the specific place of infection cannot be ascertained by our method since migrants do not necessarily migrate only once in a lifetime [25]. Furthermore, migrants may travel back and forth between their country of destination and country of origin. Regardless, it is critical to understand that migrants are at ongoing risk of HIV infection post-migration, and appropriate prevention services should be developed.

Over half of the migrants were diagnosed with a CD4^+^ T-cell count below 350 cells/µl. This indicates that, regardless of where HIV was acquired, migrant communities may be particularly vulnerable to late diagnosis and consequently short-term mortality within one year of diagnosis and morbidity in addition to facilitating onward viral transmission. This situation is exacerbated in places where migrants are unaware of, or unfamiliar with, the national healthcare service [26], have language difficulties and/or because of stigma, both perceived or real, including the fear that migrants will be reported to the authorities [27]. It is not surprising that migrants who live longer in their destination country are more likely to acquire HIV post-migration. Further work is needed to ensure prevention services are available for all migrant populations, regardless of recency of arrival.

Our study has highlighted three important findings. Firstly, the risk of HIV acquisition post-migration is not limited to long-term residents; almost one in three migrants who were diagnosed 1–5 years after migration were estimated to have acquired HIV post-migration. Secondly, MSM as well as heterosexual migrants were at similarly high risk of acquiring HIV infection post-migration. Thirdly, while the risk of migrants from high prevalence countries acquiring HIV post-migration is higher, we show that migrants from within Europe are also at risk from acquiring HIV post-migration.

Overall, our estimates are in agreement with previously published studies [11] in Sweden [28] and Italy [29], and comparable to estimates among heterosexual migrants in the UK [9]. However, our study has several new aspects, however. Compared with the aMASE study, our study is not reliant on a qualitative interview, the population studied is 10-fold larger, and we include trend data and provide more accurate timing of infection through adjustment for seroconversion. Also in contrast to the other past studies, we have developed an approach that can be feasibly reproduced in many settings because the method relies on routinely collected surveillance data. We strongly recommend the routine collection of CD4^+^ T-cell count at diagnosis, country of birth and year of arrival to allow estimation of post-migration HIV acquisition across countries.

Our trend data indicate that the proportion of migrants who acquired HIV infection post-migration increased in the UK (from 38% in 2007 to 49% in 2014) and Belgium (from 26% in 2007 to 33% in 2014). The proportion dropped slightly in the 2 most recent years in both countries; this may be due to a decrease in HIV transmission in MSM observed in both countries [30,31]. In addition, in the UK, the decline in post-migration acquisition after 2014 could also be due to fewer migrants from high HIV prevalence countries (specifically from sub-Saharan Africa) arriving in the UK [32]. In Sweden and Italy, the number of HIV diagnoses among migrants increased over the past decade, despite a decreasing trend in the proportion acquired post-migration [4].

The uncertainties related to the application of CD4^+^ T-cell count at diagnosis to modelled slopes of CD4^+^ T-cell decline have been described previously [9,33]. A single CD4^+^ T-cell count cannot provide reliable information on the length of infection for an individual since CD4^+^ T-cell counts are affected by many factors including sleep [34], exercise [35], diet [36] and other infections [37]. However, our analysis is conducted at the population level so the extent of the variation becomes less apparent at this scale. Furthermore, our model represents an enhancement on previous methods. We used a large cohort of HIV-positive individuals (1,653) with 15,881 CD4^+^ T-cell counts; these data are unlikely to be replicable in the future with wide-scale adoption of treatment at diagnosis. The accuracy was enhanced further by restricting the inclusion criteria to people with a negative test within 1 year of diagnosis (compared with 3 years [22,23,38,39]).

To attenuate the impact of seroconversion [20] on CD4^+^ T-cell trajectories and to limit inherent bias in assuming that the seroconversion date is the mid-point of the HIV negative and positive dates, we applied an anchor date 3 months after diagnosis and included consecutive CD4^+^ T-cell counts thereafter. Our estimates of CD4^+^ T-cell trajectories fall within the range of published models [20,22,23,37,38,40,41]. Nevertheless, interpretation should take the IQR into account.

CD4^+^ T-cell trajectories are only based on UK and Swedish data with an MSM cohort (diagnosed earlier than heterosexual people, on average). While MSM largely constituted the total number of seroconverted individuals, no significant differences were found in the median CD4^+^ T-cell count at the anchor date and in the decline by exposure, indicating that the slope can be considered representative for heterosexuals. Because of the comparatively small number of counts among people who inject drugs (PWID), this group was excluded from the analyses. While our model is still valid in countries with concentrated epidemics among PWID, the model would have to be specifically parametrised to PWID populations in these settings.

We took a conservative approach assigning the 5,984 migrants whose infection was probably acquired in the same year as their arrival in the category of acquiring infection pre-migration. If these individuals were instead assigned as acquiring infection post-migration, the overall estimate of migrants with post-migration HIV acquisition would increase from 40% to 65%. 

We excluded approximately half of the newly diagnosed migrants due to missing information. The completeness of the ‘year of arrival’ variable among migrants was around 70% in the UK, Belgium and Sweden and lower in Italy, which significantly increased over time to 76% in 2016. There were no major differences in the demographic or clinical characteristics of people included/excluded from the analysis (Supplement). However, those excluded from the analysis were slightly more likely to be MSM, arrived more recently and had a higher CD4^+^ T-cell count at diagnosis; this indicates that we may be underestimating the proportion of people acquiring HIV post-migration. Thus, we believe our estimates, especially estimates in more recent years, are applicable to the overall national reported migrant population.

While our method has largely attenuated the impact of seroconversion in the modelled CD4^+^ T-cell trajectories, it is possible that a small number of migrants were diagnosed during seroconversion; this potential bias would underestimate post-migration acquisition. However, the likelihood of migrants being diagnosed during seroconversion is low. A recent UK study found that only 6% (108/1,728) of patients with a CD4^+^ T-cell count at diagnosis may feasibly have had their CD4^+^ T-cell count taken during seroconversion (based upon the last negative test and tests for recently acquired infection [42]). Potentially, other information can also help to assign the timing of infection in relation to migration, such as a previous negative HIV test date and primary infection. This information was not incorporated since only the UK and Sweden have information on previous HIV tests; of these, only a small proportion of migrants reported previous HIV negative tests. While the IQR around the estimate of post-migration acquisition indicates the extent of uncertainty, it should not be interpreted as CI. The number and proportion of migrants who were first diagnosed in the country of birth before being diagnosed post-migration cannot be captured; such individuals are more likely to have been allocated as acquiring infection pre-migration if they had a low CD4^+^ T-cell count at presentation in their destination country. Alternatively, they could have been miscategorised as post-migration if they received treatment before migration and if they had a low CD4^+^ T-cell count at diagnosis in the destination country. This may have ’inflated’ the denominator and the proportion who acquired infection post-migration may be even higher. Finally, results may be affected by differential testing and screening policies for migrants in each setting.

## Conclusion

Although the estimated proportion of post-migration HIV acquisition among migrants aged 15 years and older differed across countries and risk groups, our results deliver a consistent message that migrants are at continued risk of acquiring HIV infection after arrival to the destination country. We postulate that this is probably the case in other European countries where migrants from high HIV prevalence settings arrive and settle. Our findings underscore the need to target appropriate HIV prevention activities among migrant populations, regardless of recency of arrival, age and sexual orientation. Furthermore, HIV prevention messages aimed at migrants must be effectively tailored and delivered alongside HIV testing. The barriers to HIV testing, including language and cultural barriers, poverty, affordability of testing, treatment and care regardless of residency status must be addressed for efforts to be effective.

## Supplementary Data

1760000 Supplement
