# Supplementary material for: Post-migration acquisition of HIV: Estimates from four European countries, 2007 to 2016
Source: Euro Surveill. 2021 Aug 19;26(33):2000161. doi: 10.2807/1560-7917.ES.2021.26.33.2000161 (PMC8380976; doi:10.2807/1560-7917.ES.2021.26.33.2000161)
Supplement: Supplement [file 20-00161_BROWN_Supplement.pdf]

"This supplementary material is hosted by Eurosurveillance as supporting information alongside the article Post migration acquisition of HIV: Estimates from four European countries, 2007 to 2016" on behalf of the authors who remain responsible for the accuracy and appropriateness of the content. The same standards for ethics, copyright, attributions and permissions as for the article apply. Eurosurveillance is not responsible for the maintenance of any links or email addresses provided therein

Supplementary information

Modelling CD4 trajectories to estimate time of HIV seroconversion

1. <u>Population</u>

We used a cohort of adults (aged 15 years and over) with a known last negative and first positive HIV test in one year, who had longitudinal CD4 counts extracted from the new HIV diagnoses surveillance databases from the UK and Sweden to model CD4 projections from seroconversion to HIV diagnosis. Both data sets comprise nationally comprehensive data with multiple CD4 counts for every individual. To eliminate the potential bias of temporarily low CD4 counts occurring shortly after HIV seroconversion, CD4 counts within 91 days of HIV diagnosis were excluded and an "anchor" date was set at 91 days post diagnosis (Figure 1).

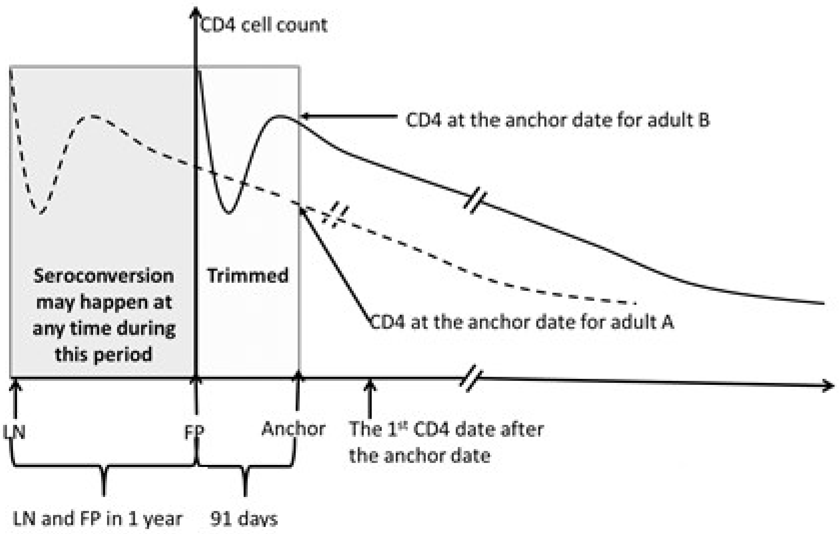

----- Adults A had HIV seroconversion shortly after the Last Negative date

___Adults B had HIV seroconversion shortly before the First Positive date

Figure 1. Timeline since the last negative HIV testing

The eligibility criteria were:

i. a documented HIV negative test result within one year of HIV diagnosis, and
ii. at least two CD4-cell measurements before treatment initiation and/or death taken after the anchor date (91 days after HIV diagnosis) and within ten years from diagnosis.

2. Methods

CD4 slopes were modelled using the CD4 counts that were reported from the anchor point onwards. In order to normalise the marker distribution, the square root of all CD4 counts was undertaken; this transformation linearized CD4 change over time[1;2]. For every individual, the time between the anchor date (91 days after HIV diagnosis) and subsequent CD4 counts was calculated.

A linear regression model was run to calculate the slope; the intercept was taken as the estimated first CD4 count taken after the anchor date. Multilevel linear regression models with random effects on the square root of CD4 cell count were run for specific population subgroups to identify potential factors (including age at HIV diagnosis, sex, ethnicity, HIV exposure, world region of birth and HIV subtype (UK only) that significantly impact on the intercept and slope. For region of birth, three groups were defined (Europe, Africa and Other) since smaller breakdowns would not yield sufficient population sizes and most patients were born in Europe and Africa within this population,

The distributions (the median and interquartile ranges) of the estimated intercepts were summed for each population subgroup and CD4 decline slopes adjusted for the significant factors identified.

Thus, the median time between the anchor date and the first CD4 was calculated as follows:

t-median (probable median interval between the anchor date and first CD4)

$$= \frac{\sqrt{M} - \sqrt{first\ CD\ \ \ cell\ count}}{S}$$

where M is the median of the intercept, and S is the slope.

As the anchor date is 91 days (0.25 years) after the first positive HIV testing date and can be up to 1.25 years after the last negative HIV testing date (Figure 1), and seroconversion might happen anything between a person's last negative and first positive HIV testing, the person's seroconversion date was between 1.25 – 0.25 years before the anchor date. For each adult, the following formulae were used to estimate the upper, central and lower time (in years) between the HIV seroconversion date and the first available CD4 cell count that occurred after the anchor date and prior to ART initiation.

t-upper 25 percentile (the probable longest time length between HIV seroconversion and an adult's first CD4 cell count)

$$= \frac{\sqrt{U} - \sqrt{first\ CD\ \ \ cell\ count}}{S_b + S_r + S_a(age\ at\ diagnosis)} + 1.25 \qquad \text{(formula 1)}$$

t-central (a central estimate of the time length between HIV seroconversion and an adult's first CD4 cell count)

$$= \frac{\sqrt{M} - \sqrt{first\ CD4\ cell\ count}}{S_b + S_r + S_a(age\ at\ diagnosis)} + 0.75 \qquad \text{(formula 2)}$$

t-lower 25 percentile (the probable shortest time length between HIV seroconversion and an adult's first CD4 cell count)

$$= \frac{\sqrt{L} - \sqrt{first\ CD4\ cell\ count}}{S_b + S_r + S_a(age\ at\ diagnosis)} + 0.25 \qquad \text{(formula 3)}$$

Where U, M and L are upper interquartile limit, median and lower interquartile limit of the intercept (estimated CD4 at the anchor date), S_b is the CD4 decline speed per year for the baseline group (people born in Europe aged 15 years at diagnosis), S_r CD4 decline speed per year adjusted world region of birth and S_a CD4 decline speed per year adjusted by age.

3. Results

3.1 Estimates of CD4 slope and CD4 value at anchor date

A total of 1,653 adults with last negative and HIV diagnosis dates within one year were included (1,233 from the UK and 420 from Sweden). This constituted 15,881 CD4-cell counts. Most were men (90%) and born in Europe (84%). The median interval between the last negative and first positive test was 191 days (IQR: 105 - 271), with a median of 141 days (IQR: 115 - 187) between HIV diagnosis and the first CD4-cell count after the anchor date. The median interval from the anchor date to end of follow-up was 3.7 years (IQR: 1.5 - 7.0).

Overall estimates of the intercept and slope are provided in Table 1. In univariate analysis of the combined UK-Swedish data, age at diagnosis and world region of birth (Europe, African and Other regions) were significantly associated with the slope and world region of birth was significantly associated with the intercept. Sex and exposure to HIV were not found to be significantly associated and were dropped from the multilevel linear regression models while age at diagnosis and world region of birth were included (Table 1).

Table 1: Square root of CD4 cell count at HIV infection and CD4 cell decline by age and world region of birth (UK and Sweden)

		**coefficient (95% CI)**	**p value**
**Square root of CD4 cell count at time origin (Intercept)** †	Baseline*	23.852 (23.036, 24.669)	<0.001
	Age at diagnosis	-0.008 (-0.031, 0.015)	0.500
	World region of birth		
	Europe	Ref	
	Africa	-2.2598 (-3.510, -1.685)	<0.001
	Other	-0.387 (-1.144, 0.371)	0.317
**Square root of CD4 cell count decline per year (Slope)** †	Baseline*	-1.062 (-1.381, -0.742)	<0.001
	Age at diagnosis	-0.014 (-0.024, -0.005)	0.002
	World region of birth		
	Europe	Ref	
	Africa	0.543 (0.179, 0.907)	0.003
	Other	0.069 (-0.234, 0.372)	0.654

* Adults aged 15 years born in Europe are baseline group.

† Age at HIV diagnosis was a continuous variable in the final model. Coefficients obtained from multivariate analysis show adults in older age-groups had a faster rate of CD4 decline compared to those in younger groups.

$$\sqrt{CD4_{ij}} = \beta_0 + \beta_1 * regionofbirth_i + \beta_2 * time_{ij} + \beta_3 * regionofbirth_i * time + \beta_4 * age_i + \beta_5 * age_i * time + r_{i0} + r_{i1} * time_{ij} + \epsilon_{ij}$$

Where $\sqrt{CD4_{ij}}$ is the jth CD4 cell count (on square root scale) of the ith individual, $time_{ij}$ is the time interval between the anchor date and the jth. The CD4 date (with day as the unit of time) $\boldsymbol{\beta}$ are fixed effects: β_0 was the fixed intercept, β_1 was the fixed intercept adjusted by world region of birth, β_2 was the fixed coefficient of time, β_3 was the fixed coefficient of time adjusted by world region of birth, β_4 was the fixed intercept adjusted by age, β_5 was the fixed coefficient of time adjusted by age. $\boldsymbol{r}$ are random effects: r_{i0} was the random intercept for the ith. individual, r_{i1} was the random coefficient for the

ith. individual. ε_{ij} were the residual errors. The covariance matrix for the random effects was unstructured.

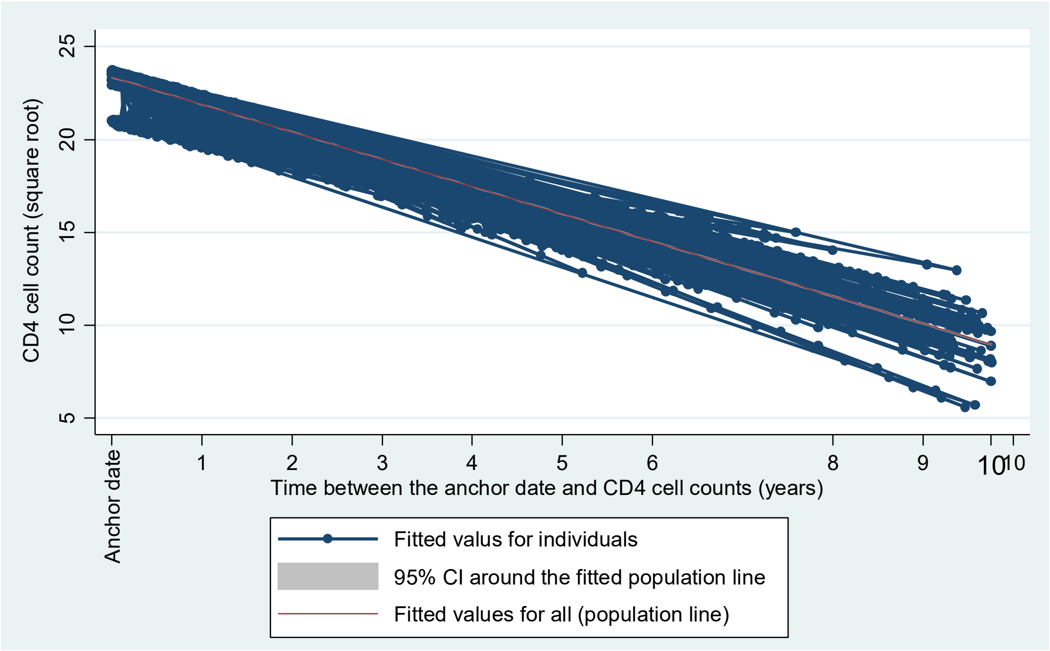

Figure 2. Fitted CD4 cell decline (square root) from the anchor date

3.2 Estimates of CD4 slopes and CD4 count value at anchor date by Country of birth

In the UK-Sweden study, median and interquartile for the first CD4 cell counts after the anchor date were 561 cells/mm^3 (IQR: 444 - 690) among people born in Europe, 438 cells/mm^3 (IQR: 363 - 570) among those born in Africa, and 543 cells/mm^3 (IQR: 434 - 699) among those born elsewhere. Figure 3 shows the median CD4 trajectories after the anchor date by region of birth for selected ages.

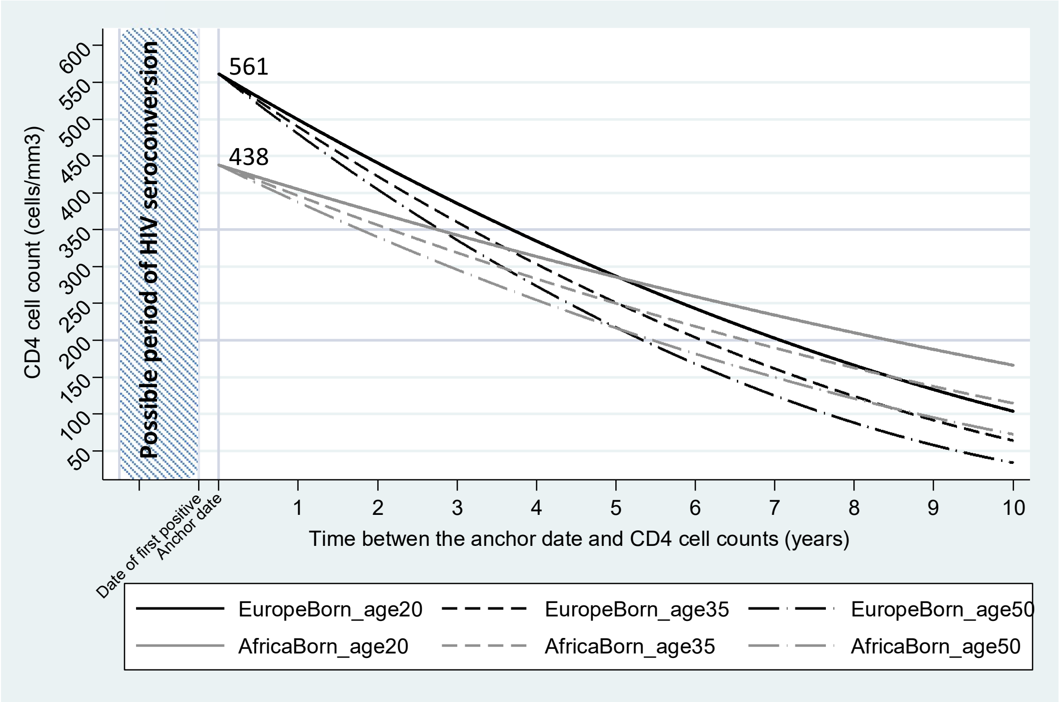

Figure 3: Median CD4 cell decline by world region of birth and age at HIV diagnosis Table 2 shows the model adjustments by country of birth and age. These estimates can be applied to a newly diagnosed individual with a date of HIV diagnosis and CD4 count at diagnosis to estimate a period of seroconversion according to region of birth and age.

The baseline group (aged 15 years at diagnosis; born in Europe) had a median CD4 cell count of 569 cells/mm3 (95%CI: 531 – 609) after the anchor date. On average, CD4 cell count dropped 1.062 cells/mm3 (95%CI: 1.381 – 0.742) on the square root scale each year; this translates to an average of 50 cells/mm^3 in the first year following the anchor date, 47 cells/mm^3 in the second year and 45 cells/mm^3 in the third year.

The time between the median of date of seroconversion (t-central in the formula 2) to first CD4 date was 5.0 (IQR: 1.1 – 9.6). Migrants in Belgium had a short time lag (3.8, IQR: 0.75 – 8.2) compared to those from the other three countries (UK (5.1, IQR: 1.2 – 9.8), Sweden (5.3, IQR: 1.8 – 9.3), Italy (5.1, IQR: 1.3 – 10.5).

Table 2. Parameters and estimated values (combined UK-Swedish data)

**Parameters**	**World region of birth**			
	Europe	Africa	Other areas	Unknown
Intercept - Upper interquartile limit (U)	690	570	689	703
- Median (central) intercept (M)	561	438	543	559
- Lower interquartile limit (L)	444	364	434	439
CD4 decline speed per year for the baseline group (S_b)	1.062	1.062	1.062	0.941
CD4 decline speed per year adjusted world region of birth (S_r)	0	-0.543	-0.069	0
CD4 decline speed per year adjusted by age (S_a)	0.014	0.014	0.014	0.015

3.3 Sensitivity analyses

Four sensitivity analyses were conducted to test the model robustness in relation to estimating the proportion of post-migration acquisition. First, we reset the anchor point from three months to six weeks after HIV diagnosis. Secondly, we used five-year age bands (instead one-year bands. Thirdly, we adopted the mid-point of the last negative and first positive dates as the estimated infection date. Finally, we investigated the impact of HIV subtype on the CD4 trajectory among UK adults with date last negative and HIV diagnosis within one year (complete for 70%). Results from the sensitivity analyses were similar to results presented above (data not shown).

References

(1) Klein MB, Young J, Dunn D, Ledergerber B, Sabin C, Cozzi-Lepri A et al. The effects of HIV-1 subtype and ethnicity on the rate of CD4 cell count decline in patients naive to antiretroviral therapy: a Canadian-European collaborative retrospective cohort study. *CMAJ Open* 2014; 2(4):E318-E329.

(2) Lodi S, Phillips A, Touloumi G, Geskus R, Meyer L, Thiebaut R et al. Time from human immunodeficiency virus seroconversion to reaching CD4+ cell count thresholds <200, <350, and <500 Cells/mm(3): assessment of need following changes in treatment guidelines. *Clin Infect Dis* 2011; 53(8):817-825.

(3) Kulkarni H, Okulicz JF, Grandits G, Crum-Cianflone NF, Landrum ML, Hale B et al. Early postseroconversion CD4 cell counts independently predict CD4 cell count recovery in HIV-1-postive subjects receiving antiretroviral therapy. *J Acquir Immune Defic Syndr* 2011; 57(5):387-395.

(4) Rodriguez B, Sethi AK, Cheruvu VK, Mackay W, Bosch RJ, Kitahata M et al. Predictive value of plasma HIV RNA level on rate of CD4 T-cell decline in untreated HIV infection. *JAMA* 2006; 296(12):1498-1506.

(5) Concerted Action on Seroconversion to AIDS and Death in EuropeCollaboration. Differences in CD41 cell counts at seroconversion and decline among 5739 HIV-1 infected individuals with known dates of seroconversion. *J Acquir Immune Defic Syndr* 2003; 34:76-83.

(6) Wolbers M, Babiker A, Sabin C, Young J, Dorrucci M, Chene G et al. Pretreatment CD4 cell slope and progression to AIDS or death in HIV-infected patients initiating antiretroviral therapy--the CASCADE collaboration: a collaboration of 23 cohort studies. *PLoS Med* 2010; 7(2):e1000239.

(7) Holmes CB, Wood R, Badri M, Zilber S, Wang B, Maartens G et al. CD4 decline and incidence of opportunistic infections in Cape Town, South Africa: implications for prophylaxis and treatment. *J Acquir Immune Defic Syndr* 2006; 42(4):464-469.
